# Metabolomic Analysis of the Ameliorative Effect of Enhanced Proline Metabolism on Hypoxia-Induced Injury in Cardiomyocytes

**DOI:** 10.1155/2020/8866946

**Published:** 2020-11-26

**Authors:** Jiacheng Wang, Zhimin Xue, Chunting Hua, Jun Lin, Zhida Shen, Yinjing Song, Hangying Ying, Qingbo Lv, Meihui Wang, Binquan Zhou

**Affiliations:** ^1^Department of Cardiology, Sir Run Run Shaw Hospital, Zhejiang University School of Medicine, Hangzhou, China; ^2^Key Laboratory of Cardiovascular Intervention and Regenerative Medicine of Zhejiang Province, Hangzhou, China; ^3^Department of Dermatology, Sir Run Run Shaw Hospital School of Medicine Zhejiang University, Hangzhou, China

## Abstract

**Background:**

Coronary heart disease is currently the leading cause of death in humans. Its poor prognosis and high mortality are associated with myocardial ischemia, which leads to metabolic disorder-related cardiomyocyte apoptosis and reactive oxygen species (ROS) production. Previous cardiovascular metabolomics studies in humans and mice have shown that proline metabolism is severely altered after cardiomyocyte hypoxia. Proline dehydrogenase (PRODH) is located on the inner mitochondrial membrane and is an enzyme that catalyzes the first step of proline catabolism, which plays an important role in improving the cellular redox state. In vitro oxygen-glucose deprivation can mimic in vivo myocardial ischemic injury. This study is aimed at investigating whether enhancing proline metabolism by overexpressing PRODH can ameliorate hypoxia-induced injury in cardiomyocytes and to reveal the related altered metabolites and mechanistic pathway via untargeted metabolomics analysis.

**Methods and Results:**

First, through public database analysis and RT-qPCR and western blot analyses in a cardiomyocyte hypoxia model, we found that the expression of the proline-degrading enzyme PRODH was downregulated after myocardial infarction and hypoxia exposure. Second, LDH assays, terminal deoxynucleotidyl transferase dUTP nick end labeling (TUNEL), DHE staining, flow cytometric apoptosis analysis with DCFH and Annexin V-FITC/PI, and western blot analysis were used to assess the injury level in cardiomyocytes. Enhanced proline metabolism induced by PRODH overexpression reduced the levels of reactive oxidative stress and apoptosis, whereas PRODH knockdown had the opposite effects. Third, untargeted metabolomics analysis revealed that the protective effect was associated with significant changes in metabolism linked to sphingolipid signaling pathways, unsaturated fatty acid biosynthesis, phosphocreatine, glutathione disulfide, aminoacyl-tRNA biosynthesis, and ABC transporters.

**Conclusions:**

Our study demonstrated a protective effect of enhanced proline metabolism in cardiomyocytes under hypoxia, providing a novel strategy for exploring new treatments for coronary heart disease.

## 1. Introduction

According to the most recently updated American Heart Association (AHA) statistics, the overall prevalence of cardiovascular disease (CVD) in the United States among adults is 48.0%, and coronary heart disease (CHD)—a group of diseases including stable angina, unstable angina, myocardial infarction (MI), and sudden cardiac death—is the leading cause (43.2%) of death from CVD [1]. Myocardial ischemia, characterized by the interruption of blood and oxygen flow to the myocardium, can trigger excessive reactive oxygen species (ROS) production and a significant increase in cardiomyocyte apoptosis [2]. Although many treatments for CHD are available, such as early revascularization, *β*-blockers, statins, and angiotensin-converting enzyme inhibitors, these treatments provide limited symptomatic relief, and the discovery of new therapeutic strategies for myocardial ischemia is urgently needed.

Metabolic disorders, termed “metabolic remodeling,” caused by cardiomyocyte hypoxia during CHD are the causes of cardiomyocyte apoptosis and ROS production [3]. Transcriptomics and proteomics can predict future events, but metabolomics can reveal past events in an organism. This approach, which allows comprehensive profiling of small molecular substances in biological systems, is increasingly being applied to investigate CVD mechanisms and potential new treatment targets [4]. Previous cardiovascular metabolomics studies in humans and mice have shown that proline metabolism is severely altered after hypoxia in cardiomyocytes [5, 6]. Proline and its metabolism impact cell survival and death outcomes by influencing the cellular redox state and maintaining cellular energy under oxidative and nutrient stress conditions, contributing to the tricarboxylic acid cycle and glutathione (GSH) biosynthesis [7].

Proline dehydrogenase (PRODH) is located on the inner mitochondrial membrane and is an enzyme that catalyzes the first step of proline catabolism [8]. Under the catalysis of PRODH, two electrons are transferred from proline to the flavin cofactor to generate 1-pyrroline-5-carboxylic acid (P5C) and reduced flavin. Simultaneously, flavin adenine dinucleotide (FAD) is reduced to FADH2, which can be used in oxidative phosphorylation to generate ATP [9]. After P5C is spontaneously converted to gamma-glutamate semialdehyde (GSA), using nicotinamide adenine dinucleotide as the electron acceptor, P5C dehydrogenase (P5CDH) oxidizes GSA to L-glutamic acid [10]. The glutamic acid produced by the oxidation of proline enters the tricarboxylic acid cycle after being converted into *α*-ketoglutarate. Oxidation of a proline molecule can produce approximately 30 equivalents of ATP, which provide vital energy for cells [7].

In addition, previous studies have shown that PRODH involves in mitochondrial redox regulations and plays an important role in protecting cancer cells against hydrogen peroxide-mediated oxidative stress damage [8]. However, the changes in intracellular metabolism under hypoxia after enhancement of proline metabolism by PRODH overexpression have not been reported. Therefore, whether enhancing proline metabolism by overexpressing PRODH can improve the redox state of cardiomyocytes with hypoxic damage is an urgent question to be answered. In this study, we used untargeted metabolomic analysis to reveal related altered metabolites and pathways after enhancement of proline metabolism in cardiomyocytes under hypoxia.

## 2. Materials and Methods

### 2.1. Cell Culture and Hypoxia Model Establishment

The rat cardiomyocyte cell line H9c2 was purchased from the American Type Culture Collection (ATCC), and cells at passage 3-8 were used. H9c2 cells were maintained in DMEM supplemented with 10% FBS at 37°C in a humidified atmosphere of 5% CO_2_. Cells were subjected to experimental procedures at 80%-90% confluence. To mimic ischemic injury in vitro, the OGD model was employed. In brief, H9c2 cells were incubated in glucose-free/serum-free DMEM and exposed to a hypoxic environment containing 94% N_2_, 5% CO_2_, and 1% O_2_ for 16 h.

### 2.2. Adenoviral Infection Experiments

A recombinant adenoviral vector overexpressing rat PRODH (Gene ID: 680409) and an adenovirus expressing a small hairpin RNA (shRNA) sequence (Ad-shPRODH) targeting rat PRODH were successfully constructed by Shanghai GenePharma Biomedical Technology (Shanghai, China). We also constructed a control adenoviral vector encoding green fluorescent protein that did not include the PRODH coding sequence, and this construct was used as a negative control (Ad-NC). Similarly, a scrambled shRNA construct was designed and synthesized and was used as a negative control (Ad-shNC). The sequence of the shRNA against rat PRODH was 5′-GGACTATGGTGTGGAGGAA-3′, and the sequence of the negative control (Ad-shNC) sequence was 5′-GTTCTCCGAACGTGTCACGT-3′, which had no significant homology to known genes. H9c2 cells at 50% confluence were infected with Ad-PRODH, Ad-NC, Ad-shPRODH, or Ad-shNC (MOI = 100) for 12 h and were then used for further experiments.

### 2.3. Analysis of LDH Leakage

Cell injury was assessed by the biomarker LDH. At the end of incubation, LDH release into the culture supernatant was measured at 490 nm with a commercial LDH kit (CK12, Dojindo, Japan) according to the manufacturer's instructions.

### 2.4. Apoptosis Assay

Apoptosis was detected with an Annexin V-FITC/PI Apoptosis Detection Kit (KGA108, KeyGen, China) and TUNEL using an In Situ Cell Death Detection Kit (Roche, USA) according to the manufacturer's instructions as we described previously [11].

### 2.5. Measurement of Intracellular ROS

ROS levels in H9c2 cells were measured by flow cytometry with the fluorescent probe DCFH-DA (10 *μ*M) (Sigma, USA, D6883) as we described previously [11]. The fluorescence probe DHE was used to measure intracellular superoxide anion levels. Cultured H9c2 cells were incubated with 10 *μ*M DHE (Yeason, China, 50102ES02) and 1X Hoechst 33342 (Beyotime Biotechnology, China, C1029) for 30 min. Then, the cells were washed with serum-free DMEM 3 times to remove background fluorescence and were observed by fluorescence microscopy.

### 2.6. Dataset Analysis

The RNA-seq dataset GSE46224 contains mRNA expression levels in the cardiac tissues of 8 nonfailing patients and 8 ischemic HF patients (https://www.ncbi.nlm.nih.gov/geo/query/acc.cgi?acc=GSE46224) [12]. We compared the RPKM values of the proline-degrading enzyme PRODH between the 8 nonfailing patients and 8 ischemic HF patients or 8 nonischemic HF patients. The RNA-seq dataset GSE114695 contains mRNA expression levels in LV tissues of mice in the 1-day, 1-week, and 8-week MI groups and the sham group (https://www.ncbi.nlm.nih.gov/geo/query/acc.cgi?acc=GSE114695) [13]. We compared the RPKM values of the proline-degrading enzyme PRODH between the sham and 1-day, 1-week, and 8-week MI groups.

### 2.7. Untargeted Metabolomic Analysis

Detailed methodology of LC-MS/MS analysis and data processing can be found in the previous study [14]. For statistical analysis of metabolomics results, after normalization to the total peak intensity, the processed data were uploaded into before being imported into SIMCA-P (version 14.1, Umetrics, Umea, Sweden) and MetaboAnalyst (https://www.metaboanalyst.ca/) [15], where they were subjected to multivariate data analysis, including Pareto-scaled PCA and OPLS-DA. Sevenfold cross-validation and response permutation testing were used to evaluate the robustness of the model. The VIP value of each variable in the OPLS-DA model was calculated to indicate its contribution to the classification. Metabolites with a VIP value of >1 were further subjected to Student's *t*-test at the univariate level to measure the significance of each metabolite, and *p* values of less than 0.05 were considered statistically significant. Based on multivariate analysis and the original MS spectra, the discriminating metabolites were identified by comparison with the human metabolome database (http://www.hmdb.ca). Pathway analyses were performed using MetaboAnalyst and KEGG (http://geneontology.org/). In the enrichment analysis of the KEGG pathway annotations of the target metabolite set, the KEGG pathway was taken as a unit, and all metabolites in each pathway were taken as the background, and the distribution of each KEGG pathway in the target metabolite set and the total metabolite set was accurately evaluated by Fisher's exact test to evaluate the significance level of the enrichment of a metabolite in a KEGG pathway. Metabolite cluster analysis (clustering) was performed as follows: first, the quantitative information of the target protein set was normalized (with an interval of (-1)). Second, Cluster3.0 software was used to simultaneously classify the two dimensions of sample and protein expression (distance algorithm: Euclid, connection mode: Average linkage). Finally, the hierarchical clustering heat map was generated in Java TreeView software.

### 2.8. RNA Isolation and Real-Time PCR

A detailed methodology can be found in our previous description [11]. The sequences of the primers were as follows: *β*-actin (forward, 5′-AAGTCCCTCACCCTCCCAAAAG-3′, reverse, 5′-AAGCAATGCTGTCACCTTCCC-3′) and PRODH (forward, 5′-GCCAGTGACGGTGGTTTTTC-3′, reverse, 5′-CATCTTGGCGATGCTCTCCT-3′).

### 2.9. Western Blot Analysis

A detailed methodology can be found in our previous description [11]. Primary antibodies against the following proteins were used in the present study: *β*-actin (#100118, GeneTex), PRODH (#22980-1-AP, Proteintech), and cleaved caspase 3 (#19677-1-AP, Proteintech).

### 2.10. Statistical Analysis

One-way ANOVA or Student's *t*-test was applied to determine the statistical significance of differences in GraphPad Prism 8.0. All results are expressed as the mean ± SD values. Differences with a *p* value of less than 0.05 were considered statistically significant. The number of independent experiments performed is indicated in the figure legends.

## 3. Results

### 3.1. The Expression of the Proline-Degrading Enzyme PRODH Is Downregulated after MI and Hypoxia

**Figure 1:**
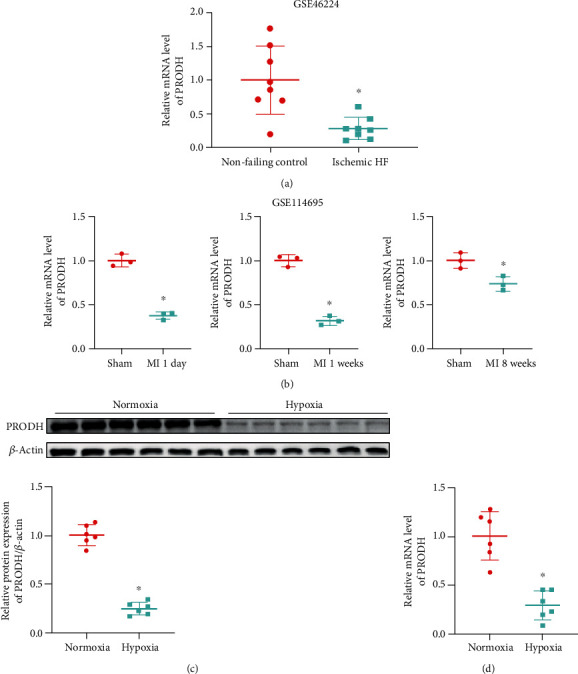
The expression of the proline-degrading enzyme PRODH is downregulated after MI and hypoxia. (a) Relative expression levels of the proline-degrading enzyme PRODH in 8 nonfailing patients and 8 ischemic HF patients from dataset GSE46224 (*n* = 8). (b) Relative expression levels of the proline-degrading enzyme PRODH between the sham and 1-day, 1-week, or 8-week MI groups from dataset GSE114695 (*n* = 3). (c) Western blot analysis and quantification of PRODH expression in the normoxia control group and the hypoxia group (*n* = 6). (d) Results of quantitative statistical analysis of PRODH expression as measured by RT-qPCR in the normoxia control group and the hypoxia group (*n* = 6). The data are presented as the mean ± SD values. ^∗^*p* < 0.05 versus the normoxia control group.

The RNA-seq dataset GEO46224 contains mRNA expression levels of cardiac tissues from 8 nonfailing patients and 8 ischemic heart failure (HF) patients (https://http://www.ncbi.nlm.nih.gov/geo/query/acc.cgi?acc=GSE46224) [12]. We compared the reads per kilobase million mapped reads (RPKM) values of the proline-degrading enzyme PRODH between the 8 nonfailing patients and 8 ischemic HF patients. The expression fold changes and statistical analysis results showed that the expression of the proline-degrading enzyme PRODH decreased significantly after ischemic HF (Figure 1(a)). The RNA-seq dataset GEO114695 contains mRNA expression levels from left ventricle (LV) tissues of MI or sham mice. We compared the RPKM values of the proline-degrading enzyme PRODH between the sham group and the 1-day, 1-week, and 8-week MI groups [13]. The expression fold changes and statistical analysis results showed that the expression of the proline-degrading enzyme PRODH decreased significantly after MI (Figure 1(b)).

To mimic ischemic injury in vitro, the oxygen-glucose deprivation (OGD) model was employed. OGD injury was induced by incubating H9c2 cells with glucose-free DMEM and exposing them to a hypoxic environment containing 94% N_2_, 5% CO_2_, and 1% O_2_ for 16 h. The protein and mRNA expression levels of the proline-degrading enzyme PRODH were dramatically decreased in H9c2 cardiomyocytes after hypoxic injury (Figures 1(c) and 1(d)), indicating that the proline-degrading enzyme PRODH might be involved in mediating hypoxic injury in cardiomyocytes.

### 3.2. Enhanced Proline Metabolism Induced by Overexpression of PRODH Reduces Apoptosis Levels, whereas PRODH Knockdown Has the Opposite Effect

**Figure 2:**
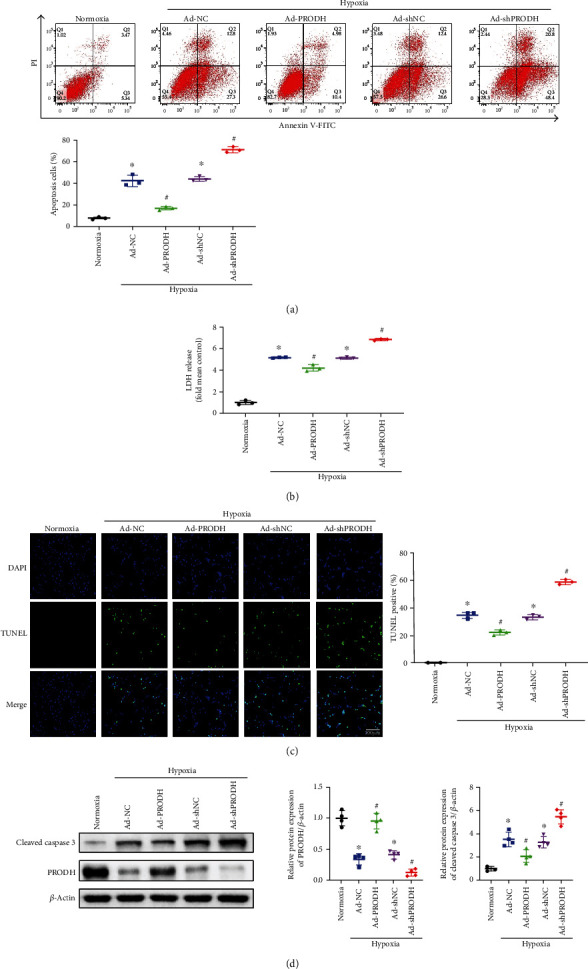
Enhanced proline metabolism induced by the overexpression of PRODH reduces apoptosis levels, whereas PRODH knockdown has the opposite effect. (a) Representative images and analysis of FITC-Annexin V/PI-positive apoptotic H9c2 cells by flow cytometry (*n* = 3). (b) Relative LDH release (*n* = 3). (c) Cardiomyocyte apoptosis was evaluated by TUNEL, and the percentage of TUNEL-positive cells is shown. Scale bar, 100 *μ*m (*n* = 3). (d) Representative images and quantitative analysis of cleaved caspase-3 expression in H9c2 cells (*n* = 4). The data are presented as the mean ± SD values. ^∗^*p* < 0.05 versus the normoxia control group; ^#^*p* < 0.05 versus the Ad-NC or Ad-shNC negative control group.

Cardiomyocyte apoptosis is an essential element associated with myocardial hypoxia-induced injury. Therefore, flow cytometric analysis was employed to evaluate H9c2 cardiomyocyte apoptosis. Quantitative analysis of flow cytometry data confirmed that compared to the proportion of apoptotic cells in the normoxia control group, the proportion of apoptotic cells was significantly increased after hypoxic injury, while PRODH overexpression in H9c2 cardiomyocytes markedly inhibited this increase. Conversely, knockdown of PRODH in H9c2 cardiomyocytes increased the proportion of apoptotic cells (Figure 2(a)). As lactate dehydrogenase (LDH) release is a recognized marker of cell injury, the release of LDH into the culture medium was also investigated. Compared to that in the control group, LDH release was significantly increased after hypoxic injury, while PRODH overexpression in H9c2 cardiomyocytes markedly inhibited the release of LDH. Conversely, knockdown of PRODH in H9c2 cardiomyocytes increased the release of LDH (Figure 2(b)). Similarly, compared to the number of TUNEL-positive cells in the normoxia control group, the number of TUNEL-positive cells was significantly increased after hypoxic injury, while PRODH overexpression in H9c2 cardiomyocytes markedly inhibited this increase. In contrast, knockdown of PRODH in H9c2 cardiomyocytes resulted in a marked increase in the number of TUNEL-positive cells (Figure 2(c)). Moreover, quantitative analysis confirmed that compared to normoxia control group, the protein expression of cleaved caspase-3, a marker of apoptosis, was significantly increased after hypoxic injury, while PRODH overexpression in H9c2 cardiomyocytes markedly inhibited this increase. Conversely, knockdown of PRODH in H9c2 cardiomyocytes increased the protein expression of cleaved caspase-3 (Figure 2(d)).

### 3.3. Enhanced Proline Metabolism Induced by Overexpression of PRODH Decreases Reactive Oxidative Stress, whereas PRODH Knockdown Has the Opposite Effect

**Figure 3:**
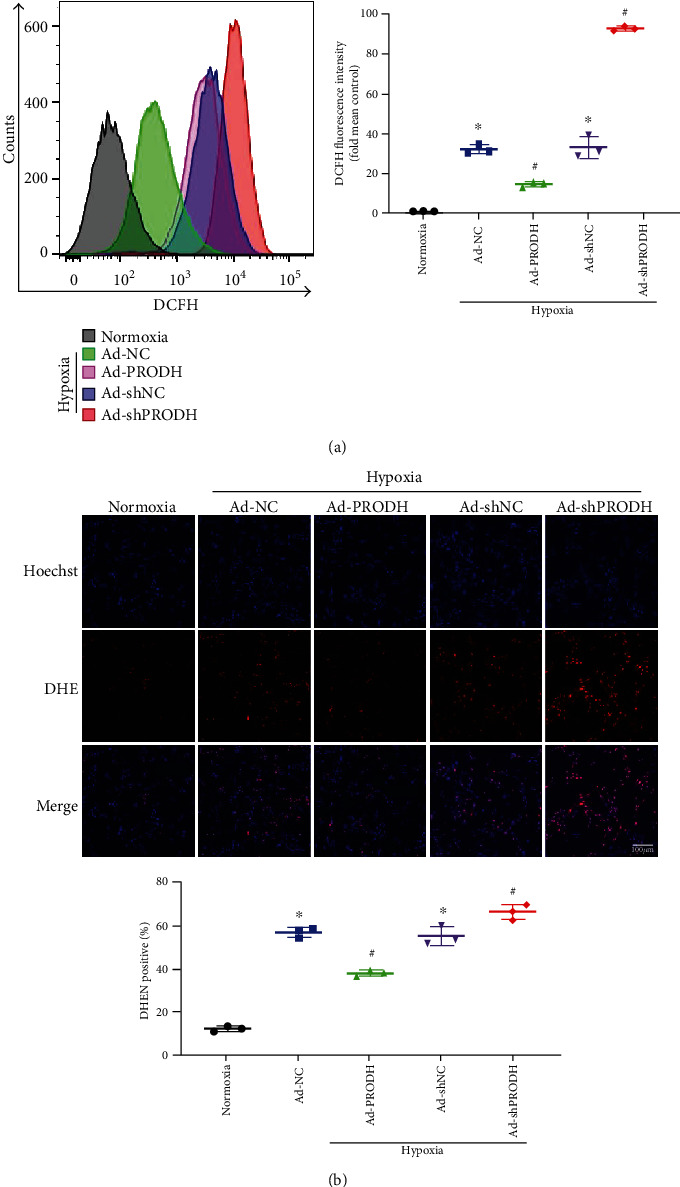
Enhanced proline metabolism induced by overexpression of PRODH decreases reactive oxidative stress, whereas PRODH knockdown has the opposite effect. (a) Representative images and quantitative analysis of reactive oxidative stress as assessed by flow cytometric analysis with DCFH fluorescent staining (*n* = 3). (b) Representative images and quantitative analysis of superoxide production as measured by DHE fluorescence. Scale bar, 100 *μ*m (*n* = 3). The data are presented as the mean ± SD values. ^∗^*p* < 0.05 versus the normoxia control group; ^#^*p* < 0.05 versus the Ad-NC or Ad-shNC negative control group.

ROS are the key executors of oxidative stress, which induces cardiomyocyte apoptosis during ischemia and hypoxia. Thus, we assessed ROS levels by 2′,7′-dichlorodihydrofluorescein diacetate (DCFH-DA) staining with flow cytometry and by dihydrogen ethidium (DHE) staining with fluorescence microscopy. Quantitative analysis showed that compared to those in the normoxia control group, the relative DCFH fluorescence intensity and the proportion of DHE-positive cells were increased after hypoxic injury, while PRODH overexpression in H9c2 cardiomyocytes markedly inhibited these increases (Figures 3(a) and 3(b)). Conversely, knockdown of PRODH in H9c2 cardiomyocytes markedly increased the relative DCFH fluorescence intensity and the proportion of DHE-positive cells (Figures 3(a) and 3(b)).

### 3.4. Overexpression of the Proline-Degrading Enzyme PRODH to Enhance Proline Metabolism Reprograms the Metabolism of Cardiomyocytes with Hypoxia-Induced Injury

Numerous studies have shown that cardiomyocyte hypoxia after MI leads to myocardial metabolic disorders [16], and altering the metabolism of certain substances after myocardial cell hypoxia can ameliorate this metabolic disorder and reduce cardiomyocyte injury [17, 18]. Therefore, we hypothesized that the enhancement of proline metabolism by overexpression of PRODH and the resulting reductions in cardiomyocyte apoptosis and ROS production may also be caused by improving specific aspects of cardiomyocyte metabolism. We used untargeted metabolomics analysis to reveal these potential mechanisms. In total, 10935 features in positive ion mode and 8069 in negative ion mode were identified in 10 samples from the Ad-NC group and the Ad-PRODH group.

**Figure 4:**
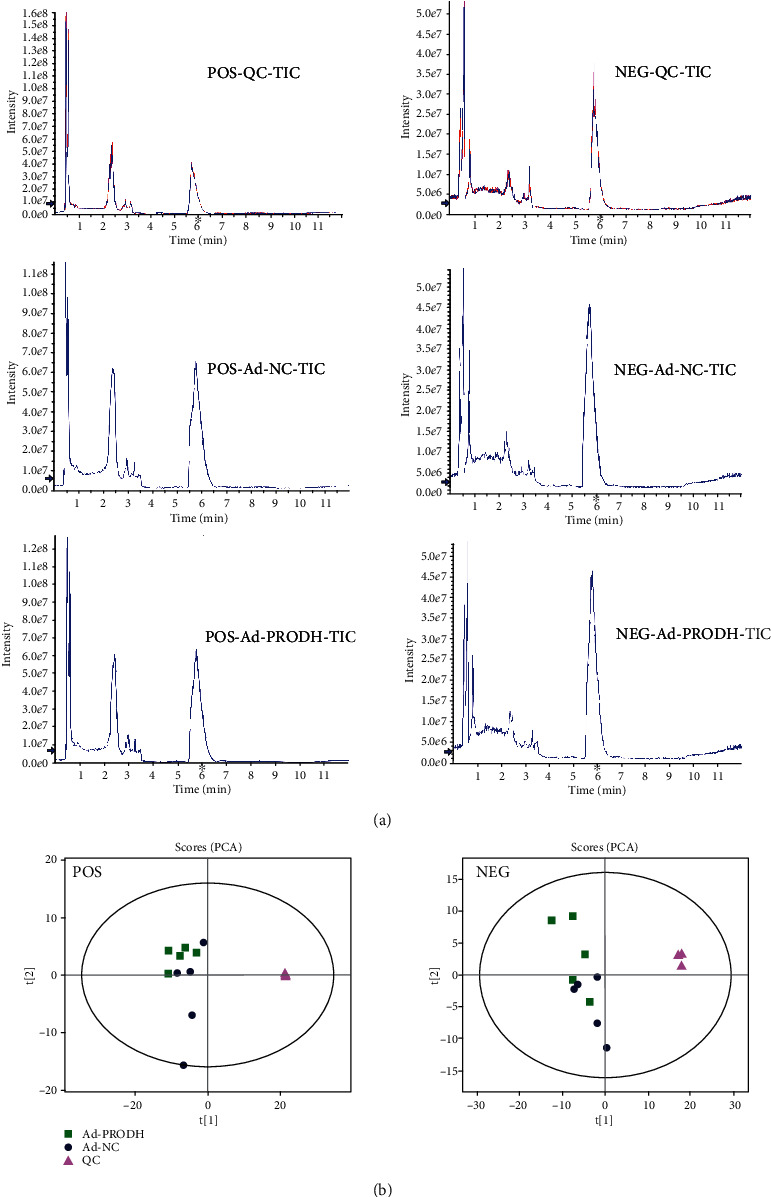
(a) Total ion chromatograms for the QC group, Ad-NC group, and Ad-PRODH group in positive and negative ion modes. (b) PCA score plot: (▲) QC group (■) Ad-PRODH group, and (●) Ad-NC group.

The representative total ion current (both positive and negative) data obtained from the cell samples in the quality control (QC) group, Ad-NC group, and Ad-PRODH group are shown in Figure 4(a). The retention time of each major chromatographic peak in the different groups, with good overlap, demonstrated the excellent stability and reproducibility of the liquid chromatography-mass spectrometry (LC-MS) system throughout the sequence. All data were analyzed using SIMCA-P software for discrimination and selection of significant variables. The principal component analysis (PCA) score plot for the QC group, Ad-NC group, and Ad-PRODH group is shown in Figure 4(b). The plot shows a trend of intragroup aggregation and intergroup separation. The high degree of aggregation in the QC group demonstrated the high stability of the LC-MS system throughout the sequence.

**Figure 5:**
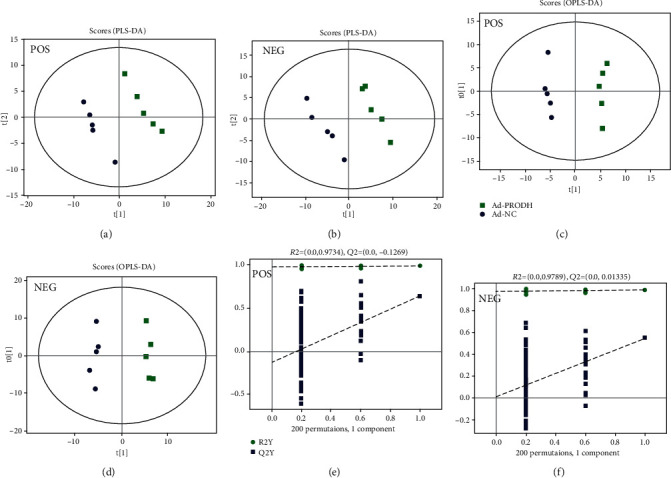
PLS-DA score plot and OPLS-DA for the model discriminating cell samples from the Ad-NC group and Ad-PRODH group. (a) POS-PLS-DA score plot. (b) NEG-PLS-DA score plot. (c, d) POS-OPLS-DA score plot. (e, f) NEG-OPLS-DA score plot: (■) Ad-PRODH group, and (●) Ad-NC group.

We further used partial least squares discriminant analysis (PLS-DA) to screen the differentially expressed metabolites between the Ad-NC group and the Ad-PRODH group. The PLS-DA scores showed a clear separation between the Ad-NC group and the Ad-PRODH group (Figures 5(a) and 5(b)). In addition, we used orthogonal partial least square discriminant analysis (OPLS-DA) for supervised data analysis to elucidate the metabolic variations. An OPLS-DA model was constructed to distinguish metabolic patterns between the Ad-NC group and the Ad-PRODH group in both positive and negative ion modes. In theory, the R2Y and Q2 values should be close to 1, which indicates a high predictive ability. As illustrated in Figures 5(c)–5(f), the metabolic profiles of cardiomyocyte samples were distinctly different between the Ad-NC group and the Ad-PRODH group.

**Figure 6:**
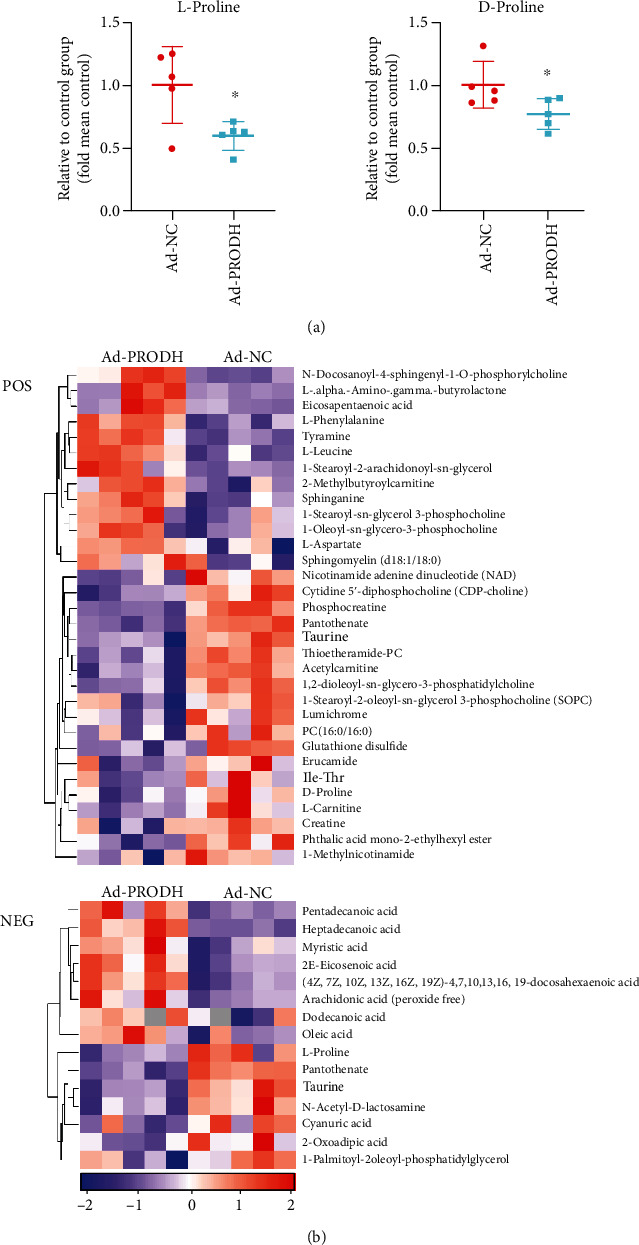
(a) Relative levels of L-proline and D-proline in the Ad-NC group and the Ad-PRODH group. (b) Heat map of the 47 differentially regulated endogenous metabolites between the Ad-NC group and the Ad-PRODH group in positive and negative ion modes. The data are presented as the mean ± SD values. ^∗^*p* < 0.05 versus the normoxia control group.

**Figure 7:**
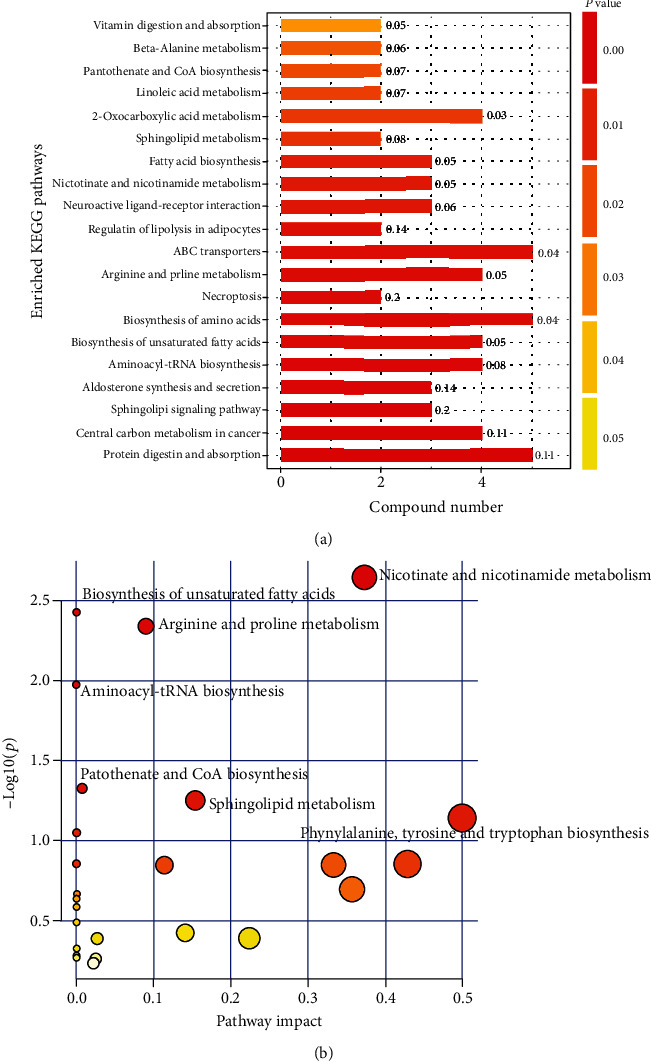
Altered metabolic pathways between the Ad-NC group and the Ad-PRODH group. (a) Metabolism-related pathways with significant changes as determined by KEGG pathway analysis. (b) Summary of pathway analysis results related to the differentially regulated metabolites generated by MetaboAnalyst.

According to the criteria for multivariate and univariate statistical significance (variable importance in projection (VIP) > 1 and *p* < 0.1), 15 metabolites were differentially expressed between the Ad-NC group and the Ad-PRODH group in negative ion mode, and 32 differentially expressed metabolites were identified in positive ion mode. These metabolites are listed in Table 1. Obviously, proline metabolism was indeed enhanced by the overexpression of the proline-degrading enzyme PRODH, and we observed significant decreases in the levels of both L-proline and D-proline (Figure 6(a)). In addition, a hierarchical clustering heat map was generated to visualize the data more intuitively. The heat map indicated that the concentrations of metabolic biomarkers in the Ad-PRODH group differed from those in the Ad-NC group (Figure 6(b)). Then, pathway analysis using the Kyoto Encyclopedia of Genes and Genomes (KEGG) database showed that the metabolites that were significantly altered between the Ad-NC group and the Ad-PRODH group were highly associated with ABC transporters, aminoacyl-tRNA biosynthesis, biosynthesis of unsaturated fatty acids, arginine and proline metabolism, sphingolipid signaling pathways, fatty acid biosynthesis, and so on (Figure 7(a)). Furthermore, to reveal the metabolic processes involving these metabolites, the significantly altered metabolites were entered into MetaboAnalyst 4.0 (https://www.metaboanalyst.ca/) for enrichment pathway analysis [15]. According to the fold enrichment values of the pathways, the major modulated pathways involved biosynthesis of unsaturated fatty acids, arginine and proline metabolism, aminoacyl-tRNA biosynthesis, sphingolipid metabolism, sphingolipid metabolism, and so on (Figure 7(b)).

## 4. Discussion

Despite advancements in percutaneous coronary intervention (PCI) and drug therapy over the past decades, CHD remains a leading cause of morbidity and mortality and, indeed, is a worldwide epidemic. Hypoxic injury is the primary cause of ROS production and apoptosis in cardiomyocytes [2]. Accumulating evidence suggests that metabolic remodeling resulting from myocardial ischemia is the primary reason for the poor prognosis of CHD, and advancing the understanding of metabolic alterations occurring in CHD patients is quite urgent to improve the prognosis of patients with CHD [19]. In this study, for the first time, liquid chromatography-tandem mass spectrometry- (LC-MS/MS-) based untargeted metabolomics analysis was performed to reveal the pathways associated with metabolic alterations after enhancement of proline metabolism in an H9c2 cardiomyocyte OGD model, which was utilized to mimic the ischemic injury observed in vivo. Via processing and statistical analysis of the metabolomics data, we discovered a variety of metabolites and metabolic pathways related to hypoxic injury in cardiomyocytes that were altered after enhancement of proline metabolism.

Sphingolipids are a class of lipids that are major components of eukaryotic cell membranes, which are biologically essential for maintaining cell structure and function, as well as cell growth, survival, and apoptosis [20]. Recent studies have proven that sphingolipids are released during ischemia and hypoxia in the human myocardium [21] and that this release may promote the protection of myocardial cells against ischemic injury [22, 23]. In this study, we observed that sphinganine (VIP = 1.3672, FC = 1.4180, *p* = 0.0015) and other related products of phospholipid metabolism were altered significantly when proline metabolism was enhanced in cardiomyocytes under hypoxia, suggesting that enhanced proline metabolism may play a protective role through regulation of phospholipid metabolism.

In addition, cardiomyocytes with enhancement of proline metabolism under hypoxia exhibited increased synthesis of fatty acids, especially unsaturated fatty acids such as eicosapentaenoic acid (EPA) (VIP = 4.1471, FC = 1.9963, *p* = 0.0422). Changes in lipid metabolism during myocardial ischemia can cause changes in membrane fluidity, permeability, or signaling cascades and exert complex effects on the physiological functions of the heart. EPA, a very-long-chain n-3 fatty acid, is highly unsaturated and increases membrane fluidity. Through modulation of the physical properties of membranes, EPA provides a specific environment to support the function of membrane proteins, such as receptors, transporters, ion channels, and signaling enzymes [24]. To date, substantial evidence has accumulated from prospective and case–control studies indicating that a higher intake of EPA is associated with a lower risk of adverse CVD outcomes in populations [25, 26]. The conclusion that very-long-chain n-3 fatty acids have a role in reducing the risk of CVD, especially CHD, is fully supported by the American Heart Association [27].

Phosphocreatine (P-Cr) is a high-energy phosphoric acid compound found in muscles or other excitatory tissues (such as brain and nerves) and is a temporary storage form of high-energy phosphoric acid groups [28]. When phosphocreatine is hydrolyzed, 10.3 kcal of free energy is released per mole of compound, which is more than the amount of energy released by ATP (7.3 kcal per mole). Under pathological conditions where energy production is impeded, such as hypoxia, phosphocreatine rapidly transfers its phosphate group to ADP, thus reconstituting the ATP store that could not otherwise be replenished due to hypoxia [29]. Our study showed that the enhancement of proline metabolism after hypoxia significantly reduced the level of phosphocreatine (VIP = 1.8213, FC = 0.4369, *p* = 0.00007), indicating that the cardiomyocyte phosphocreatine/creatine system prevents or delays the exhaustion of the ATP store that would otherwise occur because of the lack of oxidative glycolysis consequent to hypoxia. In addition to the level of phosphocreatine, the level of glutathione disulfide (VIP = 1.6194, FC = 0.4546, *p* = 0.0132) was also decreased after enhancement of proline metabolism under hypoxia. Glutathione and its oxidized form is one of the most important redox buffer pairs in the cell [30]. On the one hand, it can directly eliminate ROS, and at the same time, it has an important regulatory effect on cellular ROS signal transduction during oxidative stress [7]. Different studies have shown that enhanced proline metabolism increases the level of reduced glutathione in cells and reduces that of glutathione disulfide. Our research also confirms this event, which is one of the mechanisms by which intracellular ROS production decreases after enhancement of proline metabolism under hypoxia.

Aminoacyl-tRNA biosynthesis, which is key for delivering amino acids to the ribosome and ensuring the accuracy of translation, was significantly altered after enhancement of proline metabolism under hypoxia in cardiomyocytes [31]. The latest clinical evidence suggests that aminoacyl-tRNA biosynthesis plays a key role in maintaining left ventricular diastolic function, which opens new research perspectives for the treatment and early prevention of HF [32]. Our study also showed that the ABC transporter pathway was changed significantly after the enhancement of proline metabolism. ABC transporters are a family of ATP-dependent transporters that can transport a variety of endogenous compounds, including amino acids, ions, nucleotides, lipids, and peptides, across the cell membrane. Researchers have found that ABC transporters are altered after MI and are involved in cardiac homeostasis [33, 34], which constitute future research directions regarding the cardioprotective effect of proline metabolism.

The limitation of this study is that although the OGD hypoxic injury model was used to simulate the in vivo ischemic state, metabolism is a process of systemic changes in the organism. The future clinical therapeutic application of enhanced proline metabolism requires additional experiments to prove the effectiveness of this approach in vivo. Another limitation of this study was that only an untargeted semiquantitative MS approach was used to screen differential metabolites. Thus, further verification with a targeted quantitative method is required. Based on the discussion of the above-untargeted metabolomics results, we will conduct further verification and functional studies on the basis of these significantly changed metabolites and metabolic pathways to clarify the possible mechanism by which enhanced proline metabolism protects cardiomyocytes against hypoxic damage and to explore new treatments for ischemic heart disease.

## 5. Conclusions

In summary, our study demonstrated a protective effect of enhanced proline metabolism in cardiomyocytes under hypoxia. First, we found that the expression of the proline-degrading enzyme PRODH was downregulated after MI and hypoxia. Second, we demonstrated that enhanced proline metabolism induced by overexpression of PRODH reduced reactive oxidative stress and apoptosis levels, whereas PRODH knockdown had the opposite effects. Third, untargeted metabolomics analysis revealed that the protective effect was associated with significant changes in metabolism associated with sphingolipid signaling pathways, unsaturated fatty acid biosynthesis, phosphocreatine, glutathione disulfide, aminoacyl-tRNA biosynthesis, and ABC transporters. The changes discussed above provide insight into novel mechanisms by which enhanced proline metabolism protects cardiomyocytes against hypoxic injury and support the exploration of these mechanisms to design new therapeutic approaches for CHD.

## Figures and Tables

**Table 1:** Statistical analysis of 47 differential metabolites from the comparison of the Ad-NC group and the Ad-PRODH group under hypoxia in cardiomyocyte.

Ion mode	Description	m/z	rt(s)	VIP	FC	*p* value
Positive	1,2-dioleoyl-sn-glycero-3-phosphatidylcholine	786.60	143.49	7.05	0.90	6.59*E*-04
	1-Stearoyl-2-arachidonoyl-sn-glycerol	627.53	113.65	6.55	2.27	9.58*E*-03
	2-Methylbutyroylcarnitine	246.17	242.30	1.05	1.59	7.77*E*-03
	Acetylcarnitine	204.12	315.31	3.30	0.72	7.71*E*-04
	Cytidine 5′-diphosphocholine (CDP-choline)	489.11	449.49	1.19	0.66	1.33*E*-03
	D-Proline	116.07	323.98	2.54	0.77	4.70*E*-02
	Eicosapentaenoic acid	369.17	32.89	4.15	2.00	4.22*E*-02
	Glutathione disulfide	613.16	573.61	1.62	0.45	1.32*E*-02
	L-.alpha.-Amino-.gamma.-butyrolactone	102.05	89.49	4.18	5.89	4.38*E*-02
	L-Aspartate	134.04	421.99	1.48	1.72	3.27*E*-02
	L-Carnitine	162.11	387.09	1.70	0.66	2.55*E*-02
	L-Leucine	132.10	323.23	1.85	1.26	5.45*E*-04
	L-Phenylalanine	166.09	322.60	1.64	1.26	3.08*E*-04
	Lumichrome	243.09	62.21	1.01	0.88	2.51*E*-02
	N-Docosanoyl-4-sphingenyl-1-O-phosphorylcholine	809.65	118.37	2.21	2.91	9.87*E*-04
	Nicotinamide adenine dinucleotide (NAD)	664.12	491.16	1.82	0.37	6.03*E*-03
	Pantothenate	220.12	278.37	3.10	0.52	6.44*E*-06
	PC (16 : 0/16 : 0)	756.55	144.77	1.37	0.94	3.98*E*-02
	Phosphocreatine	212.04	445.88	1.82	0.44	6.92*E*-05
	Phthalic acid mono-2-ethylhexyl ester	279.16	31.75	4.76	0.88	4.17*E*-03
	Sphinganine	302.30	125.24	1.37	1.42	1.46*E*-03
	Taurine	126.02	297.31	5.11	0.81	2.94*E*-03
	Thioetheramide-PC	758.57	144.77	5.66	0.91	7.10*E*-04
	Tyramine	120.08	322.71	2.08	1.25	2.95*E*-04
	1-Methylnicotinamide	137.07	256.68	2.60	0.76	8.53*E*-02
	1-Oleoyl-sn-glycero-3-phosphocholine	544.34	182.66	1.70	1.19	9.05*E*-02
	1-Stearoyl-2-oleoyl-sn-glycerol 3-phosphocholine (SOPC)	788.62	37.07	2.47	0.93	5.96*E*-02
	1-Stearoyl-sn-glycerol 3-phosphocholine	568.34	184.81	1.35	1.21	6.54*E*-02
	Creatine	132.08	348.95	1.64	0.61	5.78*E*-02
	Erucamide	338.34	33.13	4.86	0.86	9.98*E*-02
	Ile-Thr	233.15	52.99	1.02	0.60	8.87*E*-02
	Sphingomyelin (d18:1/18 : 0)	731.60	120.65	1.50	1.14	8.86*E*-02
Negative	2E-Eicosenoic acid	309.28	47.05	2.95	1.31	4.19*E*-03
	2-Oxoadipic acid	141.02	228.32	6.48	0.47	4.34*E*-02
	(4Z,7Z,10Z,13Z,16Z,19Z)-4,7,10,13,16,19-Docosahexaenoic acid	327.23	35.15	5.34	1.64	8.52*E*-04
	Arachidonic acid (peroxide free)	303.23	47.62	9.91	1.66	2.94*E*-02
	Heptadecanoic acid	269.25	35.37	2.03	1.36	2.93*E*-04
	L-Proline	114.06	324.79	1.12	0.60	2.44*E*-02
	Myristic acid	227.20	35.15	2.95	1.19	3.11*E*-02
	N-acetyl-D-lactosamine	442.15	370.14	1.06	0.70	1.06*E*-02
	Oleic acid	281.25	48.74	11.43	1.27	4.97*E*-02
	Pantothenate	218.10	279.08	3.31	0.38	1.25*E*-05
	Pentadecanoic acid	241.22	51.09	3.28	1.28	4.64*E*-03
	Taurine	124.01	297.53	4.03	0.75	1.23*E-*03
	1-Palmitoyl-2-oleoyl-phosphatidylglycerol	747.52	51.23	1.10	0.87	8.74*E*-02
	Cyanuric acid	128.01	59.06	1.72	0.59	5.57*E*-02
	Dodecanoic acid	199.17	6.28	2.18	1.78	9.90*E*-02

## Data Availability

The datasets used and analyzed during the current study are available from the corresponding author upon reasonable request.
